# Supplementary material for: Drug Transport across Porcine Intestine Using an Ussing Chamber System: Regional Differences and the Effect of P-Glycoprotein and CYP3A4 Activity on Drug Absorption
Source: Pharmaceutics. 2019 Mar 21;11(3):139. doi: 10.3390/pharmaceutics11030139 (PMC6471532; doi:10.3390/pharmaceutics11030139)
Supplement: Supplementary file 1 [file pharmaceutics-11-00139-s001.pdf]

pharmaceutics

# Supplementary Material: Drug Transport across Porcine Intestine Using an Ussing Chamber System: Regional Differences and the Effect of P-Glycoprotein and CYP3A4 Activity on Drug Absorption

**Yvonne E. Arnold, Julien Thorens, Stéphane Bernard and Yogeshvar N. Kalia**

**Table S1.** UHPLC-MS/MS methods.

(a) The training set of passively absorbed drugs.

| Compound | Chromatographic Conditions | | | | | Mass Spectrometry Conditions | | | | | |
|---|---|---|---|---|---|---|---|---|---|---|---|
| | Mobile Phases | | Col. Temp. (°C) | Flow Rate (mL/min) | Retention Time (min) | Capillary Voltage (kV) | Cone Voltage (V) | Collision Energy (eV) | MRM Transition (m/z) | ESI | LOQ (nM) |
| | Aqueous | Organic | | | | | | | | | |
| **BCS I** | | | | | | | | | | | |
| 1) Antipyrine | 40% A | 60% G | 45 | 0.2 | 0.83 | 3.7 | 6 | 20 | 189.13 > 55.47 | +ve | 17.10 |
| 2) Ketoprofen | 30% B | 52.5% G 17.5% H | 22 | 0.3 | 0.62 | 3.3 | 7 | 20 | 253.06 > 208.96 | −ve | 55.12 |
| 3) Metoprolol | 60% C | 40% G | 40 | 0.2 | 1.20 | 3.5 | 6 | 20 | 268.10 > 159.07 | +ve | 13.82 |
| 4) Propranolol | 50% C | 50% I | 55 | 0.2 | 1.28 | 3.6 | 37 | 21 | 260.07 > 116.09 | +ve | 1.34 |
| **BCS II** | | | | | | | | | | | |
| 5) Carbamazepine | 50% C | 50% I | 55 | 0.2 | 1.10 | 3.0 | 53 | 20 | 237.16 > 194.10 | +ve | 1.0 |
| 6) Naproxen | 30% D (40 mM) | 21% H 49% G | 30 | 0.2 | 0.80 | 3.0 | 7 | 20 | 231.10 > 153.15 | +ve | 13.08 |
| 7) Piroxicam | 20% E | 80% H | 30 | 0.2 | 0.90 | 0.6 | 7 | 20 | 332.13 > 94.85 | −ve | 1.5 |
| **BCS III** | | | | | | | | | | | |
| 8) Atenolol | 50% C | 50% I | 55 | 0.2 | 0.70 | 3.6 | 6 | 21 | 366.18 > 349.04 | +ve | 72.95 |
| 9) Terbutaline | 60% C | 40% H | 40 | 0.2 | 0.80 | 2.1 | 6 | 20 | 225.12 > 151.93 | +ve | 4.95 |
| **BCS IV** | | | | | | | | | | | |

| | | | | | | | | | | | |
|---|---|---|---|---|---|---|---|---|---|---|---|
| 10) Furosemide | 65% D (10 mM) | 35% H | 60 | 0.2 | 1.42 | 3.7 | 6 | 20 | 330.74 > 205.00 | −ve | 13.99 |
| 11)Hydrochlorothiazide | 20% E | 80% H | 55 | 0.2 | 0.77 | 3.7 | 6 | 20 | 297.74 > 268.90 | −ve | 3.06 |

Aqueous phases:
A: 0.1% ammonium hydroxide in $H_2O$
B: $H_2O$
C: 0.1% formic acid in $H_2O$
D: ammonium acetate in $H_2O$
E: 0.1 % acetic acid in $H_2O$
F: 2mM ammonium formate

Organic phases:
G: acetonitrile
H: methanol
I: 0.1% formic acid in acetonitrile

(b) The P-gp and CYP3A4 substrates.

| Compound | Chromatographic Conditions | | | | | Mass Spectrometry Conditions | | | | | |
|---|---|---|---|---|---|---|---|---|---|---|---|
| | Mobile Phases | | Col. Temp. (°C) | Flow Rate (mL/min) | Retention Time (min) | Capillary Voltage (kV) | Cone voltage (V) | Collision energy (eV) | MRM transition (m/z) | ESI | LOQ (nM) |
| | Aqueous | Organic | | | | | | | | | |
| **BCS I** | | | | | | | | | | | |
| Midazolam | 40% C | 60% I | 40 | 0.2 | 1.44 | 2.8 | 6 | 20 | 325.97 > 290.97 | +ve | 0.38 |
| *Hydroxymidazolam* | 40% C | 60% I | 40 | 0.2 | 1.50 | 2.8 | 6 | 20 | 342.10 > 202.99 | +ve | 1.74. |
| Verapamil | 20% A | 80% G | 45 | 0.2 | 1.30 | 3.6 | 6 | 20 | 455.29 > 149.95 | +ve | 2.64 |
| *Norverapamil* | 30% A | 70% G | 42 | 0.2 | 1.65 | 3.6 | 6 | 20 | 441.05 > 165.00 | +ve | 10.94 |
| **BCS II** | | | | | | | | | | | |
| Tamoxifen | 20% F | 80% I | 50 | 0.3 | 1.00 | 2.1 | 6 | 20 | 371.97 > 128.97 | +ve | 36.29 |
| *N-desmethyl-tamoxifen* | 20% F | 80% I | 50 | 0.3 | 2.20 | 2.1 | 6 | 20 | 388.10 > 106.93 | +ve | 32.91 |
| **BCS III** | | | | | | | | | | | |
| Cimetidine | 20% D (10 mM) | 80% G | 25 | 0.2 | 0.76 | 2.4 | 7 | 20 | 253.14 > 94.85 | +ve | 5.58 |
| Ranitidine | 30% A | 70% G | 45 | 0.2 | 0.83 | 2.4 | 6 | 20 | 315.14 > 97.82 | +ve | 2.58 |

Aqueous phases:
**A**: 0.1% ammonium hydroxide in $H_2O$
**B**: $H_2O$
**C**: 0.1% formic acid in $H_2O$
**D**: ammonium acetate in $H_2O$
**E**: 0.1 % acetic acid in $H_2O$
**F**. 2 mM ammonium formate

Organic phases:
**G**: acetonitrile
**H**: methanol
**I**: 0.1% formic acid in acetonitrile

**Table S2.** The precision and accuracy of the analytical methods.

(a) The training set of passively absorbed drugs.

| Compound | Theoretical Concentration (nM) | Experimental Concentration (nM) | Precision (%) | Accuracy (%) |
|---|---|---|---|---|
| **BCS I** | | | | |
| 1) Antipyrine | 57.37 | 62.38 ± 2.47 | 3.96 | 108.74 |
| | 286.84 | 314.57 ± 1.93 | 0.61 | 109.67 |
| | 573.68 | 571.43 ± 8.64 | 1.51 | 99.61 |
| 2) Ketoprofen | 94.38 | 96.93 ± 1.61 | 1.66 | 102.69 |
| | 245.40 | 231.38 ± 9.12 | 3.94 | 94.29 |
| | 943.84 | 1054.85 ± 10.18 | 0.96 | 111.76 |
| 3) Metoprolol | 86.70 | 94.84 ± 0.87 | 0.92 | 109.38 |
| | 216.74 | 216.79 ± 1.51 | 0.69 | 100.02 |
| | 303.44 | 289.99 ± 3.16 | 1.09 | 95.57 |
| 4) Propranolol | 12.72 | 12.37 ± 0.06 | 0.45 | 97.18 |
| | 15.91 | 16.07 ± 0.59 | 3.70 | 101.05 |
| | 25.45 | 24.97 ± 0.12 | 0.48 | 98.13 |
| **BCS II** | | | | |
| 5) Carbamazepine | 20.19 | 19.27 ± 0.33 | 1.70 | 95.44 |
| | 77.59 | 74.40 ± 0.53 | 0.72 | 95.89 |
| | 232.78 | 231.30 ± 3.30 | 1.43 | 99.36 |
| 6) Naproxen | 620 | 584.85 ± 41.79 | 7.57 | 94.14 |
| | 4954.4 | 4664.22 ± 353.05 | 1.55 | 102.40 |
| | 8989.84 | 9205.78 ± 142.49 | 7.14 | 94.33 |
| 7) Piroxicam | 39.84 | 38.23 ± 0.67 | 1.75 | 95.97 |
| | 159.35 | 144.13 ± 2.27 | 1.57 | 90.45 |
| | 677.23 | 686.55 ± 6.84 | 1.00 | 101.38 |
| **BCS III** | | | | |
| 8) Atenolol | 63.63 | 67.46 ± 3.14 | 4.66 | 106.03 |
| | 106.05 | 108.94 ± 7.46 | 6.85 | 102.73 |

| | | | | |
|---|---|---|---|---|
| | 424.19 | 422.51 ± 10.16 | 2.41 | 99.60 |
| | 48.27 | 49.48 ± 1.61 | 3.25 | 102.51 |
| 9) Terbutaline | 112.64 | 109.19 ± 3.74 | 3.43 | 96.94 |
| | 160.91 | 164.75 ± 3.67 | 2.28 | 102.39 |
| **BCS IV** | | | | |
| | 90.71 | 91.50 ± 2.04 | 2.22 | 100.88 |
| 10) Furosemide | 120.94 | 119.37 ± 1.81 | 1.52 | 98.70 |
| | 181.41 | 172.49 ± 7.02 | 4.07 | 95.08 |
| | 6.72 | 7.16 ± 0.50 | 6.95 | 106.63 |
| 11) Hydrochlorothiazide | 33.59 | 32.64 ± 1.23 | 3.77 | 97.17 |
| | 100.76 | 97.46 ± 4.38 | 4.50 | 96.72 |

(b) The P-gp and CYP3A4 substrates.

| Compound | Theoretical concentration (nM) | Experimental concentration (nM) | Precision (%) | Accuracy (%) |
|---|---|---|---|---|
| **BCS I** | | | | |
| Midazolam | 38.37 | 36.14 ± 1.08 | 2.98 | 94.19 |
| | 153.48 | 155.74 ± 4.32 | 2.78 | 101.47 |
| | 383.71 | 400.37 ± 10.27 | 2.56 | 104.34 |
| *Hydroxymidazolam* | 4.17 | 4.34 ± 0.23 | 5.22 | 104.00 |
| | 6.95 | 6.40 ± 0.04 | 0.61 | 92.14 |
| | 13.9. | 14.28 ± 0.22 | 1.51. | 102.73 |
| Verapamil | 134.73 | 132.89 ± 4.04 | 3.04 | 98.63 |
| | 263.97 | 251.05 ± 8.16 | 3.25 | 95.10 |
| | 527.94 | 512.58 ± 13.02 | 2.54 | 97.09 |
| *Norverapamil* | 397.20 | 390.77 ± 14.76 | 3.78 | 98.38 |
| | 794.41 | 755.97 ± 26.05 | 3.45 | 95.16 |
| | 1588.81 | 1600.69 ± 58.76 | 3.67 | 100.75 |
| **BCS II** | | | | |
| Tamoxifen | 121.13 | 115.23 ± 0.16 | 0.14 | 95.13 |
| | 181.69 | 185.25 ± 8.19 | 4.42 | 101.95 |
| | 242.25 | 242.81 ± 0.01 | 0.01 | 100.23 |
| *N-desmethyl-tamoxifen* | 8.39 | 8.20 ± 0.15 | 1.83 | 97.66 |
| | 16.78 | 16.83 ± 0.08 | 0.50 | 100.31 |
| | 97.91 | 101.14 ± 7.07 | 6.99 | 103.31 |
| **BSC III** | | | | |
| Cimetidine | 45.34 | 45.37 ± 1.21 | 2.67 | 100.08 |
| | 174.37 | 170.06 ± 6.49 | 3.82 | 97.83 |
| | 348.74 | 348.71 ± 14.77 | 4.24 | 99.99 |
| Ranitidine | 26.24 | 24.99 ± 0.39 | 1.55 | 95.25 |
| | 262.40 | 265.99 ± 5.87 | 2.21 | 101.37 |
| | 524.81 | 544.45 ± 9.91 | 1.82 | 103.74 |
